# Perceived barriers and attitudes toward arteriovenous fistula creation and use in hemodialysis patients in Palestine

**DOI:** 10.1080/0886022X.2020.1748650

**Published:** 2020-04-25

**Authors:** Ala O. Shamasneh, Anwar S. Atieh, Kamel A. Gharaibeh, Abdurrahman Hamadah

**Affiliations:** a Department of Internal Medicine, Faculty of Medicine, Al-Quds University, Jerusalem, Palestine; b Department of Medicine, Faculty of Medicine, Hashemite University, Zarqa, Jordan

**Keywords:** End stage renal disease, hemodialysis, arteriovenous fistula, central venous catheter, barriers

## Abstract

In the dialysis center in Ramallah, we investigated the attitudes and perceived barriers to having arteriovenous fistula (AVF) in 156 patients. The current method of HD access was AVF in 52% and central venous catheter in 47%. Perceived causes of no or delayed AVF were: patient’s refusal of AVF in 54.5%, late referral to a surgical evaluation in 31.3% and too long to surgical appointments in 14.2%. Among those who refused AVF, reasons were: concern about the surgical procedure in 42.5%, poor understanding of disease/access in 23.3%, fear of needles in 15.1%, denial of disease or need for HD in 17.8%, and cosmetic reasons in 1.4%. Forty six percent of patients believed they received education about AVF prior to the creation of HD access, and 73.7% would recommend AVF as the method of access due to the lower risk of infection (96%), easier to care for (16%), easier showering (14%), and better-associated hygiene (3%). In conclusion, the majority would recommend an AVF as the mode of vascular access for HD. The most common barrier to having an AVF was patient’s refusal to undergo AVF creation because of their concern about the surgical procedure. A systematic evaluation of the process that precedes the creation of AVF may allow for better utilization.

## Introduction

For advanced chronic kidney disease patients who will be initiated on HD therapy soon, the type of vascular HD access remains a big challenge. Guidelines from different countries strongly recommend native arteriovenous fistula (AVF) as the best method for dialysis amongst patients who are undergoing hemodialysis [1–11]. It is well established that AVF had the superiority over other types of vascular access: central venous catheter (CVC) and arteriovenous graft (AVG) since it provides the best longevity, less likely rates of infection and least association with mortality and morbidity in the majority of patients [12–15]. Despite these advantages of AVF, the number of patients undergoing dialysis through CVC or AVG is high [16]. In 2003, The Fistula First breakthrough initiative (FFBI) which was a National Access Improvement Initiative to encourage the use of AVF as vascular access in HD population. This initiative was established as a collaboration with the Centers for Medicare & Medicaid Services (CMS), the end stage renal disease (ESRD) Networks, and the entire renal community [10]. The FFBI initial goal was to increase the percentage of native AVF use to 44%, in 2009 the percentage of HD patients having AVF was 65% which exceeded the initial goal [17]. Meanwhile, the overall proportion of prevalent AVF utilization increased from 33% in all HD patients in 2003 to 62.7% by the mid of 2016 [10,16]. However, in 2015 United States Renal Data System (USRDS) annual data reported that the percentage of patients receiving HD therapy through CVC was 80% [16]. Achieving optimal AVF access is a complicated process and many barriers have been described, including hospital systems, HD patients, and and provider-related [18,20]. According to the 2017 annual health report of the Palestinian Ministry of Health, the overall number of HD patients has increased from 1014 patients in 2015 to 1119 patients in 2016 from different 12 dialysis centers in the West Bank, Palestine [18–20]. To the best of our knowledge, this is the first study in Palestine to investigate the vascular access relevant issues amongst HD patient including perceptions and barriers to AVF use. The aim of this study is to explore patients’ perceptions of advantages and perceived barriers that impede AVF utilization as a first vascular access choice.

## Material and methods

### Study design and setting

In this cross-sectional study, we investigate the attitudes toward AVF and the perceived barriers to its creation among Palestinian HD patients. We recruited all adult participants aged 18–85 years, receiving HD as outpatients from August-December of 2018 at Palestinian Medical Complex Hospital in Ramallah, Palestine which is considered one of the largest Ministry of Health dialysis units in Palestine as per the total number of patients who undergo hemodialysis weekly.

### Participants

We screened 198 participants who had the diagnosis of ESRD, undergoing regularly scheduled HD sessions of Saturday–Monday–Wednesday or Sunday–Tuesday–Thursday. Exclusion criteria included pediatric age group (less than 18 years), acute dialysis; major mental or neurological illness that precludes their ability to be recruited with fully consenting; refusal to participate; death before completing their data or those who were unavailable at the time of the study. This study was carried out in accordance with the recommendations of the Al-Quds University Research Ethics Committee with written informed consent from all subjects. The protocol was approved by the Al-Quds University Research Ethics Committee.

### Data collection

All participants underwent in-person interviews either before, after, or during the HD session using structured questions. Patients’ medical records were all reviewed to collect their demographics and characteristics information. Demographic data collected included age, sex, weight, height, body mass index (BMI). The presence of comorbidities including diabetes mellitus, hypertension, dyslipidemia, coronary artery disease, or cerebrovascular disease was recorded. Data pertaining to the cause of ESRD: Diabetes mellitus, hypertension, polycystic kidney disease, glomerulonephritis, other, or unknown were also obtained.

Information was collected regarding HD initiation, access, and attitudes toward fistula creation and use, including time in months from HD initiation, current access method and whether vein mapping was done before vascular access creation. In addition, data was gathered if patients previously received sufficient education about AVF and for those patients who did not have a fistula or had a delay in its creation, perceived barriers were explored in detail. Furthermore, patients were asked whether they recommend AVF as the preferred access to others, the reasons for their recommendation as well as the characteristics of those who refused fistula.

### Statistical methods

Data were summarized by calculating means and standard deviation (*SD*) or medians and range for quantitative variables and percentages for categorical variables. Descriptive terms were used where appropriate. The reported attitudes and perceived barriers were analyzed as categorical variables.

## Results

We screened 198 Palestinian patients who had the diagnosis of ESRD, and undergoing regularly scheduled HD therapy during the study period that extends from August to December of 2018. Out of them, 156 were included in our study and 42 were excluded (three were pediatric age group (less than 18 years); 2 refused to participate; 22 died before completing their data; and 15 were unavailable at the time of the study).

Patient’s age ranged from 18 to 85 years (*M* = 55; *SD* = 15), gender (92 males and 64 females, 59% and 41%, respectively), and 29 (19%) were smokers. Average BMI (*M* = 26; *SD* = 6). At the time of the study, patients had an average time since starting dialysis of 24 months ranged (1 to 216). Detailed demographics characteristics including the cause of ESRD and major associated comorbidities were shown in (Table 1). The current access method for hemodialysis based on age group showed that AVF is highly used in patient’s groups who are younger than 55 and between the age of 67 and 79. While, 60% of patients who are between 55 and 66 years use permanent CVC (Table 2). Patient attitudes and perceived barriers toward AVF creation are presented in Tables 3 and 4.

**Table 1.** Baseline demographics and characteristics of study participants.

Patient characteristics	Overall *n* = 156
Baseline demographics	
Age (years) mean ± *SD*	55 ± 15
Gender	
Male, *n* (%)	92 (59)
Female, *n* (%)	64 (41)
Weight (kg) mean ± *SD*	74.2 ± 16.6
Height (m) mean ± *SD*	1.66 ± 8.5
BMI (kg/m^2^) mean ± *SD*	26 ± 6
Smoker *n* (%)	29 (19)
Cause of ESRD	
Diabetes mellitus *n* (%)	68 (44%)
Hypertension *n* (%)	23 (15)%
Adult polycystic kidney disease *n* (%)	8 (5%)
Glomerulonephritis *n* (%)	21 (13%)
Other *n* (%)	19 (12%)
Unknown *n* (%)	17 (11%)
Associated comorbidities	
Diabetes mellitus *n* (%)	87 (56%)
Hypertension *n* (%)	108 (69%)
Dyslipidemia *n* (%)	60 (38%)
Coronary artery disease *n* (%)	67 (43%)
Cerebrovascular disease *n* (%)	11 (7%)
Peripheral vascular disease *n* (%)	34 (22%)

BMI: Body Mass Index; ESRD: End stage renal disease.

**Table 2.** Current access method and duration of HD based on age group.

	Age group			
	<55 years *n* = 67	55–66 years *n* = 55	67–79 years *n* = 33	≥80 years *n* = 1
Current access method				
Temporary CVC *n* (%)	1 (1.5%)	3 (5.5%)	–	1 (100%)
Permanent CVC *n* (%)	21 (31.3%)	33 (60%)	14 (42.4%)	–
AVF *n* (%)	43 (64.2%)	19 (34.5%)	19 (57.6%)	–
AVG *n* (%)	2 (3%)	0	–	–
Time in months since HD initiation, median (range)	37.9 (1–216)	25.1 (2–108)	38.6 (1–216)	–

HD: Hemodialysis; CVC: Central venous catheter; AVF: arteriovenous fistula; AVG: arteriovenous graft.

**Table 3.** Perceived barriers toward AVF creation based on age group.

	Age group			
	<55 years *n* = 55	55–66 years *n* =51	67–79 years *n* = 27	≥80 years *n* = 1
Reported outcome				
Perceived barrier to AVF^a^				
Late referral to surgical evaluation	21 (38.2%)	14 (27.5%)	7 (25.9%)	–
Refusal to undergo AVF surgery	27 (49.1%)	28 (54.9%)	17 (63%)	1 (100%)
Too long to surgical appointments after referral	7 (12.7%)	9 (17.6%)	3 (11.1%)	–

^a^Out of 134 patients with non-AVF dialysis access or delayed AVF creation.

**Table 4.** Attitudes toward AVF creation.

Reported outcome	*n* (%)
Previously received sufficient education about AVF?	
Yes	72 (46)
No	84 (54)
Previous Vein mapping done?	
Yes	87 (56)
No	69 (44)
Would you recommend AVF to other HD Patients	
Yes	115 (73.7)
No	26 (16.7)
Not reported/Not certain	15 (9.6)
If answer to above question is Yes, why would you recommend it?	
Less infection	71 (60.2)
Easier to care for	12 (10.2)
Easier showering	10 (8.5)
Better hygiene	2 (1.7)
All above	20 (16.9)
Other/unspecified	3 (2.5)

**Figure 1.**
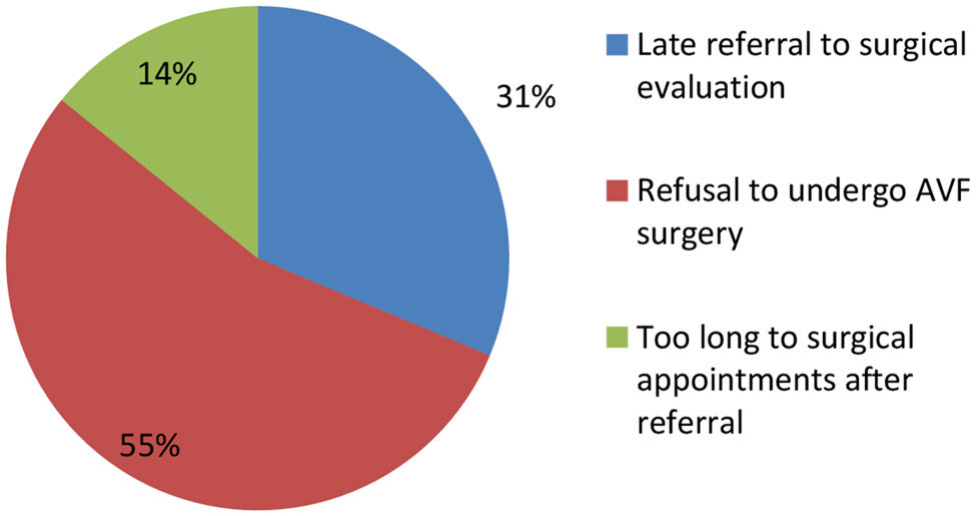
Causes of lack of AVF as dialysis access.

The most common cause for no AVF was the refusal to undergo AVF surgical procedures in 73 patients (54.5%) and there was no difference among those patients based on their age group, followed by late referral to a surgical evaluation in 42 patients (31.3%) and time too long to surgical appointments after referral in 19 (14.2%; Figure 1). Out of the patients who refused to undergo surgical procedures, 31 (42.5%) patients were concern about the surgical procedure itself, 17 (23.3%) have a poor understanding of their disease and access needs, 11 (15.1%) they fear of needles, 13 (17.8%) denial their disease or even their need for HD, and 1 (1.4%) patient due to other causes including cosmetics (Table 5).

**Table 5.** Characteristics of those who refused fistula or do not recommend it.

Characteristics of those who refused fistula	*N* (%)
Total number of those who refused/do not recommend AVF	73 (54.5)
Reason for refusing AV fistula	
Concern about the surgical procedure/refused surgery	31 (42.5)
Poor understanding of disease/access needs	17 (23.3)
Fear of needles	11 (15.1)
Denial of disease or need for HD	13 (17.8)
Others include Cosmetics reasons	1 (1.4%)

HD: Hemodialysis; AVF: arteriovenous fistula.

**Figure 2.**
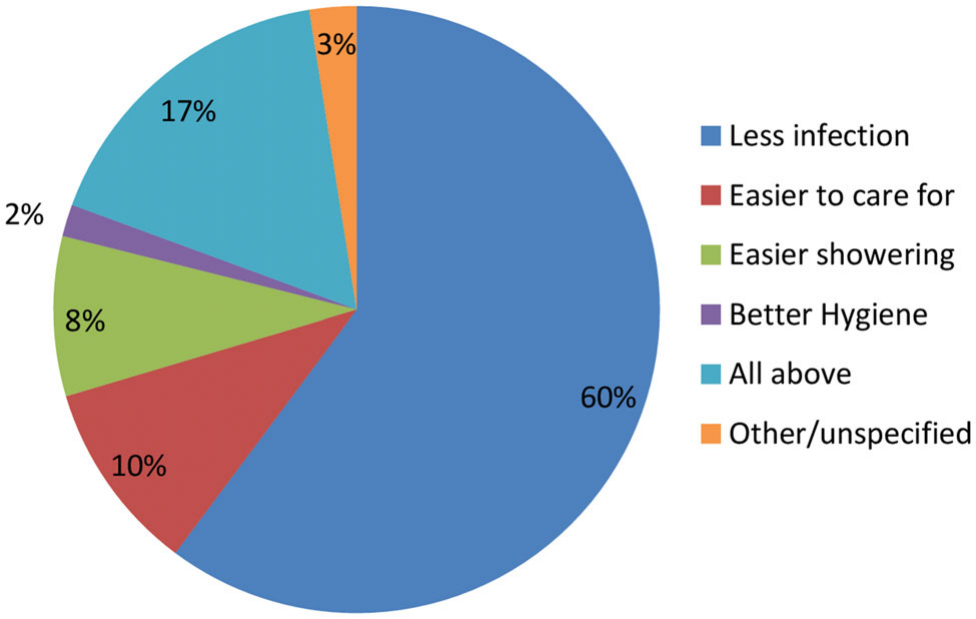
Why would you recommend AVF?

Of the overall group, 72 (46%) reported they received sufficient education and information about AVF prior to creation, on the other hand, 84 patients (54%) thought that was not the case. Vein mapping is done for 87 patients (56%) prior to an attempt for fistula creation. One hundred fifteen patients (73.7%) would strongly recommend AVF to other HD patients as a method of vascular access. Patients attributed their preferences and recommendations for AVF to many reasons including decreased risk of infection 71 (60.2%), easier to care for 12 (10.2%), emphasis on easier shower 10 (8.5%), better associated hygiene 2 (1.7%), three of the patients (2.5%) reported unspecified causes and 20 (16.9%) all of these reasons in combination (Figure 2). Overall, 26 patients (16.7%) did not recommend AVF as the method of HD access to other patients (Table 4).

## Discussion

Current clinical practice guidelines from different countries strongly advocate AVF as the best vascular access since it has been considered to have the lowest risk of complications and need for interventions, best long-term patency, and superior patient survival [21,22]. Having an AVF prior to the commencement of HD is not only associated with lower morbidity and mortality but it is also associated with better patient-reported quality of life and lower health care expenditure [25,26]. Despite this, many patients maintained on HD therapy use CVC [27]. A recent study conducted in the same area to investigate the rate of pre-dialysis nephrology care and AVF usage amongst 156 chronic HD patients showed a high incidence of CVC use and a relatively large portion of HD did not have any pre-dialysis nephrology care. Furthermore, a low incidence of AV utilization found even in patients who received pre-dialysis care [28]. Investigations regarding the system, physicians and patients' characteristics that may be responsible for delays in AVF creation remain an ongoing challenge.

To the best of our knowledge, no previous studies have presented data that help address the attitudes and perceived barriers to timely AVF creation and utilization amongst HD patients in Palestine, which is the aim of this analysis.

Our study found that most of HD patients believe that AVF is the best choice of vascular access for many reasons; the vast majority of them would recommend it to their fellow patients who are newly starting dialysis because the existing patients believe it is prone to lower risk of infection compared with CVC. Other reported advantages are related to quality of life, including easier care, better hygiene and easier for showering. These patients’ attitudes toward AVF could be attributed to their personal experience with CVC related complications in particular infection when they used it as the method of initial HD vascular access, they may have been also influenced by the experiences of other patients who suffered from the drawbacks associated with CVC.

A previous study of a national random sample of 1563 HD patients conducted in the United States to investigate the relationship of initial HD vascular access type with patient-reported health status and quality of life scores at the time of HD initiation and at day 60. Their results showed that patients with AVF at initiation and at day 60 reported perceived greater physical activity and energy, emotional and social well-being, fewer symptoms, less effect of dialysis and burden of kidney disease, and better sleep compared with patients with persistent CVC use [25]. In addition, Do Hyoung Kima et al. investigated the effects of vascular access types on the survival and quality of life and depression in the incident hemodialysis patients among 1461 patients who newly initiated HD. The primary outcomes were all-cause mortality and HRQOL and depression. The secondary outcome was all-cause hospitalization. Kidney Disease Quality of Life Short Form 36 (KDQOL-36) and Beck’s depression inventory scores were measured to assess HRQOL and depression. In the survival analysis, patients with AVF had a better survival and low hospitalization rates, and the patients with AVF or AVG showed both higher HRQOL and lower depression scores than those with CVC [29].

In another cohort study, preferences and concerns regarding HD vascular access were reviewed by asking 128 HD patients and 64 of dialysis nurses, technicians, HD access surgeons, and nephrologists, found that the access preferred by patients was of the utilization of a superficial access in the forearm which was easy to cannulate, had minimal effect on their appearance, provided quick hemostasis after dialysis and enabled arm comfort during dialysis, whereas from their point of view, the most common problem was pain during needle insertion [30].

In our study, we found that the most reported perceived barriers for those who have not been dialyzing through an AVF, or who had a delay in AVF creation were the refusal to undergo AVF surgery, late referral to surgical evaluation and too long to surgical appointments after referral.

In a study of a cohort of 319 HD patients conducted in nine nephrology centers in New Zealand and Australia, barriers to timely AVF creation were investigated. Their results revealed that lack of formal policies for patient referral, absence of patient database for access purposes that could facilitate the management, and also long wait times to surgical evaluation and access creation were the perceived barriers to access creation [31]. These barriers were previously implicated by nephrologists and primary care providers in a qualitative study to identify modifiable challenges to adequate preparation of patients for renal replacement therapy [32].

With regard to the patients who refused to undergo AVF which was the main barrier in our sample (54.5%), more details were explored to find the reasons behind their refusal. Our study revealed that concern about the surgical procedure (42.5%), poor understanding of the disease or access needs, fear of needles, denial of disease or need for HD and cosmetic reasons were the most cited barriers related to the patient. In a related systematic review of qualitative studies, aiming to understand the attitudes, beliefs, preferences, and values of 375 patients who refused AVF creation or use, Xi et al., performed interviews with those patients investigating the rationale for decision making [33]. Three main reasons that affected the decision to refuse AVF were identified: Poor previous experience with the fistula such as issues with cannulation, bleeding, or appearance, issues with knowledge transfer and informed decision making, and patient acceptance of current status quo without a desire for change. Patients can have a strong preference for the status quo and are disappointed to switch from an acute start CVC access to a long-term AVF may explain why a large number of patients in our sample refuse AVF [34]. In contrast to our study, decreasing infection rate was not the focus of the 375 patients who refused AVF creation or use in the study of Xi et al. [33]. The same was seen in another study that investigated patients’ perspectives on complications of vascular access-related interventions and found that infectious complications were not reported as a major concern by patients when the access modalities are compared. On the other hand, physical complications which manifested as fear and pain associated with cannulation were more likely a cause of patients’ dissatisfaction with AVF compared to CVC access [35]. In another study, fear of painful and difficult cannulation and patients trust in their ability to manage complications of CVC were the reasons to avoid AVF [36].

Nearly half of our patients reported they received insufficient education about different types of HD access and the pros and cons of each one. It was previously noted that patients with less dialysis knowledge were found to be less likely to use arteriovenous access for dialysis at initiation and transitioning to AVF after starting hemodialysis, since the poor understanding of the AVF is an important aspect regarding the barriers related to the patient [37]. In fact, 23% of our sample reported a poor understanding of vascular access and this is a modifiable challenge which by improving patient’s education may facilitate the AVF utilization [38]. Several factors contribute to the heterogeneity of AVF prevalent use and the distribution that include the age (young vs. old) [39]. In our study, the largest percentage of patients under the age of 55 and between 67 and 76 currently uses AVF.

## Conclusion

In this study among dialysis patients in Ramallah/Palestine, most participants would recommend an AVF as the mode of access. Barriers to AV use were found to be classified into three major categories; provider-related in which there is a late referral to surgical evaluation, system-based including too long to surgical appointments after referral, and issues pertaining to the patient who may refuse or be reluctant to undergo AVF surgery. The reasons that stand behind the patient’s refusal of AVF were: concern about the surgical procedure, poor understanding of the disease or access needs, denial of disease or need for HD, and fear of needles. These results suggest the need for a systematic evaluation of the attitudes that precede AVF creation, to identify potential targets for care improvement such as timely referral to a surgical evaluation in addition to facilitating sufficient education about HD access methods may allow for better AVF utilization in HD patients in Palestine. Furthermore, engaging patients in care planning and decision making may improve patient knowledge about treatment options and adherence.

## Geolocation information

This study conducted at the national dialysis center in Ramallah, West Bank, Palestine, which is considered one of the largest Ministry of Health dialysis units in Palestine as per the total number of patients who undergo hemodialysis weekly.
